# Scalable distributed agreement from LWE: Byzantine agreement, broadcast, and leader election

**DOI:** 10.1007/s00446-026-00519-w

**Published:** 2026-10-01

**Authors:** Rex Fernando, Yuval Gelles, Ilan Komargodski

**Affiliations:** 1 Aptos Labs, Palo Alto, CA USA; 2 School of Computer Science and Engineering, The Hebrew University of Jerusalem, Givat Ram, Jerusalem, Israel; 3 NTT Research, Sunnyvale, CA USA

**Keywords:** Distributed agreement, Scalable, Byzantine agreement, LWE

## Abstract

Distributed agreement is a general name for the task of ensuring consensus among non-faulty nodes in the presence of faulty or malicious behavior. Well-known instances of agreement tasks are Byzantine Agreement, Broadcast, and Committee or Leader Election. Since agreement tasks lie at the heart of many modern distributed applications, there has been an increased interest in designing *scalable* protocols for these tasks. Specifically, we want protocols where the per-party communication complexity scales sublinearly with the number of parties. With unconditional security, the state of the art protocols have ${\tilde{O}}(\sqrt{n})$ per-party communication and ${\tilde{O}}(1)$ rounds, where *n* stands for the number of parties, tolerating 1/3-ϵ fraction of corruptions for any ϵ>0. There are matching lower bounds showing that these protocols are essentially optimal among a large class of protocols. Recently, Boyle-Cohen-Goel (PODC ’21, Journal of Cryptology ’24) relaxed the attacker to be computationally bounded and using strong cryptographic assumptions showed a protocol with ${\tilde{O}}(1)$ per-party communication and rounds (similarly, tolerating 1/3-ϵ fraction of corruptions). The security of their protocol relies on SNARKs for NP with linear-time extraction, a somewhat strong and non-standard assumption. Their protocols further rely on a public-key infrastructure (PKI) and a common-reference-string (CRS). In this work, we present a new synchronous protocol with ${\tilde{O}}(1)$ per-party communication and rounds but relying only on the standard Learning With Errors (LWE) assumption. Our protocol also relies on a PKI and a CRS, and tolerates 1/3-ϵ fraction of corruptions, similarly to Boyle et al. Technically, we leverage (multi-hop) BARGs for NP directly and in a generic manner that significantly deviates from the framework of Boyle et al.

## Introduction

Byzantine Agreement [1, 2] is a classical problem in distributed computing whose aim is to ensure that all non-faulty nodes in a distributed system agree on the same value, even in the presence of faulty or malicious (Byzantine) behavior. This task is tightly related to other well studied distributed agreement tasks such as Broadcast, and Committee or Leader Election. All of the above are fundamental to many distributed systems, such as blockchain protocols (e.g., Algorand [3, 4]) and secure multiparty computation (MPC) protocols [5–8].

There are many natural efficiency metrics for agreement protocols, including rounds, communication, computation, and load balance. In this work we focus on two classical ones: the number of rounds of communication required to reach agreement, and the number of bits sent by each party. Optimizing these two metrics is the subject of a rich line of active research. Due to the need to support large numbers of parties, scalability has become an important goal. Ideally, we want to design protocols where the number of rounds and the per-party communication grow (much) slower than the number of parties.

In this work, we consider the synchronous communication model and assume point-to-point channels. We further consider adversaries that corrupt parties statically (after the protocol is specified but before the protocol’s execution starts). Our main result is the first protocol with $\textsf{polylog}(n)$ rounds and $\textsf{poly}(\lambda ,\log n)$ bits of communication per party, based on standard falsifiable cryptographic assumptions; throughout, we do not attempt to optimize the precise degree of the polylogarithmic factors.

### Theorem 1

(Scalable Agreement from LWE) Assume a public-key infrastructure (PKI), a common reference string (CRS), and the hardness of the Learning With Errors problem (LWE).^1^ Then, there are *n*-party protocols for Byzantine agreement, broadcast, and committee or leader election, where each of these protocols is secure against a probabilistic polynomial-time adversary that statically corrupts up to 1/3-ϵ fraction of parties for any constant ϵ>0. Furthermore, each of these protocols terminates within $\textsf{poly}(\log n)$ rounds and each party sends $\textsf{poly}(\lambda , \log n)$ bits overall, where $\lambda \in {\mathbb {N}}$ is a computational security parameter.

Our construction, in fact, is obtained using generic usage of a multi-hop non-interactive batch argument (BARG) system for NP.^2^ Such BARGs were recently constructed assuming LWE and in the CRS model by [9, 10]. Therefore, any construction of a multi-hop BARG from other assumptions, say group-based, will immediately imply the above result under the other assumptions. (We will explain what BARGs and multi-hop BARGs are below, in Sect. 2).

*Putting the result in context*: For convenience, throughout the paper, we use the notation ${\tilde{O}}(\cdot ),\tilde{\Omega }(\cdot )$ to hide poly-logarithmic factors in *n*, and ${\tilde{O}}_\lambda (\cdot )$ to additionally hide polynomial factors in λ, the computational security parameter. That is, $\textsf{poly}(\log n) \equiv {\tilde{O}}(1)$ and $\textsf{poly}(\lambda , \log n) \equiv \textsf{poly}(\lambda ,\log n)$.

With information theoretic security, the state of the art protocol (in terms of per-party communication complexity) is due to King et al. [11, 12]. They obtained an ${\tilde{O}}(1)$-round protocol where each party communicates ${\tilde{O}}(\sqrt{n})$ bits overall (tolerating 1/3-ϵ corruptions). Prior works (e.g., [1, 2, 13–15]) all had quadratic total communication. Other works that came around the same time or after [11, 12], for example [16–18], have total quasi-linear communication but they are unbalanced; i.e., few (poly-logarithmically many) parties need to communicate with everyone. A recent work [19] gave a computationally-efficient variant of the state of the art protocol of [11, 12]. In another recent work [20], a generic transformation was shown from protocols with worst-case (pessimistic) guarantees to protocols that have optimal guarantees in an honest (optimistic) execution (and same pessimistic guarantees in the worst case).

In terms of lower bounds, few barriers are known. Holtby, Kapron, and King [21] showed that in a large class of Byzantine agreement protocols there is at least one honest party that must send $\tilde{\Omega }(n^{1/3})$ messages. Gelles and Komargodski [19] showed that in the same class of Byzantine agreement protocols, there is at least one honest party that must send *or process* $\tilde{\Omega }(n^{1/2})$ messages.

Allowing cryptography and trusted setup assumptions (that invalidate both of the above-mentioned lower bounds), Boyle, Cohen, and Goel [22, 23] achieve a protocol with essentially best-possible communication: ${\tilde{O}}(1)$ rounds and per-party $\textsf{poly}(\lambda ,\log n)$ bits of communication overall. To do this they introduce a new cryptographic primitive, which they call *succinctly reconstructed distributed signatures* (SRDS), and show that it suffices for distributed agreement. The authors then instantiate SRDS using a PKI and a strong form of succinct non-interactive arguments of knowledge (SNARKs),^3^ which are known not to be constructible based on falsifiable assumptions [24, 25]. The authors also give a construction of SRDS using falsifiable assumptions, but in order to achieve this, they modify the PKI model to be much stronger and less meaningful in the context of BA. Specifically, they assume that a single trusted party generates every key pair, where it provides valid secret keys only to a small randomly and secretly chosen subset of the parties; this PKI model can be referred to as a “centrally-generated PKI.” The construction of [22, 23] in the centrally-generated PKI model *has the trusted party choose a secret, global committee, and programs knowledge of this committee into each member’s secret key*. Thus, their construction can be seen as a way to bootstrap a single BA output (i.e., the choice of a global committee) in this model to achieve more BA outputs. It is unclear, however, what this result means in the context of actually achieving a BA protocol from scratch, since as explained before, it is assumed that a trusted party programs the output of a BA protocol into the trusted setup. Note that this is very different from the standard definition of PKI, where each party publishes a public key that it is allowed to choose arbitrarily. The authors of [22, 23] pose the existence of an efficient BA protocol from falsifiable assumptions and in the non-trusted PKI model as the main open question in this line of work.

In Theorem 1 we answer this question in the affirmative, obtaining the same efficiency properties of the resulting protocol as [22, 23] (in the non-trusted PKI model) except that we rely on the standard LWE assumption instead of (strong variants of) SNARKs. Notably, LWE is both falsifiable and believed to be quantum-secure, which has made it an attractive and widely-used assumption. This gives the first agreement protocols with ${\tilde{O}}(1)$ rounds and $\textsf{poly}(\lambda ,\log n)$ per-party communication relying on standard falsifiable assumptions. Notably, we do *not* achieve our result by following their template of instantiating SRDSs using LWE; in fact, this is still an open problem. Instead, we suggest a completely different framework for achieving scalable agreement protocols utilizing the power of quorums and multi-hop aggregate signatures. Our approach is arguably much simpler and more direct. We stress that unlike the “centrally-generated PKI model,” our protocol does not require the public-key infrastructure to guarantee *any* sort of agreement. Namely, our protocol is a secure BA protocol even if a malicious party sends inconsistent public keys to different other parties. We give a detailed overview of our ideas in Sect. 2.

*On the corruption threshold*: In the classical synchronous model with oral messages (no cryptography), Byzantine agreement is possible if and only if fewer than one third of the parties are corrupted [1, 2]. With cryptographic assumptions and authenticated channels, agreement protocols can in general tolerate a minority of corruptions (i.e., t<n/2). Our protocols tolerate a (1/3-ϵ)-fraction of static corruptions because they rely on an almost-everywhere communication-tree building block in that regime. Whether one can push the corruption threshold closer to 1/2 while retaining $\textsf{poly}(\lambda ,\log n)$ per-party communication from LWE (in the standard PKI model) is left open.

*On concrete polylogarithmic factors*: Similar to other scalable agreement protocols in this line of work, the dominant (and fairly large) polylogarithmic overhead in our complexity bounds originates from the almost-everywhere agreement subroutine. Consequently, any improvement in the concrete efficiency of this primitive would immediately translate into better overall constants. An interesting direction for future work is to reduce these polylogarithmic factors, or to develop alternative techniques that circumvent this bottleneck while maintaining the same asymptotic guarantees and reliance on falsifiable assumptions.

*On the importance of relying on falsifiable assumptions*: Roughly speaking, a falsifiable assumption [24] is one that can be phrased and modeled as an interactive game between an efficient challenger and an adversary, at the conclusion of which the challenger can *efficiently* decide whether the adversary “won” the game. The assumption states that every efficient adversary has at most a negligible winning probability in the game. Most standard cryptographic assumptions are falsifiable, including general notions (e.g. one way functions, trapdoor permutations, oblivious transfer, fully homomorphic encryption, etc.) and concrete assumptions (e.g. hardness of factoring, discrete logarithms, shortest vector problem, RSA, CDH, DDH, LWE etc.). The above notion of falsifiability captures the fact that the challenger gives us an efficient process to test whether an adversarial strategy breaks the assumption. Since the introduction of this notion, falsifiability has been an important criteria of “reasonable” or “standard” assumptions.

Intuitively, assumptions that are not falsifiable are harder to reason about, and therefore we have significantly less confidence in them. SNARKs are a common examples for a primitive that is by-itself *non-falsifiable* because for a challenger to decide whether an adversary produces a proof of a false statement, it needs to decide whether an NP statement is true or false, which could be hard in general. Additionally, it is well known that it is impossible to construct (in a certain black-box manner) SNARKs from any falsifiable assumption [25], making it an assumption that we would prefer to avoid whenever possible.

## Technical overview

We consider the following goal that will turn out to suffice for all of our applications. We want to build a communication tree, where every internal node in the tree corresponds to a small set of parties and every one of the *n* leaf nodes is virtually associated with a unique party. The root node is called the supreme committee. Intuitively, every internal node is a “committee” that certifies communication from the parent of the node to its children and vice versa. At a high level, our goal is to maximize the number of internal nodes that correspond to “good” committees (in particular, containing a majority of honest parties) and also maximize the number of leaf nodes that can communicate with the root (supreme committee) by talking only with other “good” committees (this is explained more precisely below). Obviously, if the members in each node are fixed and known, then an adversary with a sufficient corruption budget can corrupt a majority of parties in many internal nodes and eventually prevent reliable communication. Thus, while the tree structure is fixed and known ahead of time, the members in each node will be selected “on the fly”. For efficiency purposes, we want each non-leaf node to contain ${\tilde{O}}(1)$ members^4^ and that each party is a member in ${\tilde{O}}(1)$ nodes. The tree will have fan-out ${\tilde{O}}(1)$ and the depth of the tree is ${\tilde{O}}(1)$.

Let ${\mathcal {H}}$ be the set of honest parties. A resulting communication tree will induce a set $K\subseteq {\mathcal {H}}$ such that for every p∈K, it holds that

1. The path from the node associated with *p* to the root is “good”, where “good” means that the majority of each node members along the path are from *K*. That is, for every node *N* along the path between leaf *p* to the root (including the root), it holds that |N∩K|>|N|/2.
2. For each node in the tree, each node member from *K* knows the members of its neighboring nodes (parent and children).

Note that in the above two properties we do not require anything for parties in ${\mathcal {H}}\setminus K$ and so it is convenient to think of them as behaving adversarially.

If we manage to generate such a tree with $K={\mathcal {H}}$, then we are essentially done. The supreme committee members (i.e., the ${\tilde{O}}(1)$ parties associated with the root node) can perform any polynomial complexity protocol to compute Byzantine Agreement, or Committee/Leader Election, and distribute the result to all the parties using the tree. Indeed, since any honest party has a direct (and short) path to the root, involving only nodes with an honest majority, it is guaranteed that it will learn the right value. The main challenge is therefore to come up with such a communication tree.

*Prior work: using the almost everywhere communication tree*: Already in the original work of [11], a communication tree as above was constructed. However, instead of achieving it with respect to $K = {\mathcal {H}}$, they achieved it with respect to some $K' \subset {\mathcal {H}}$, where $|{\mathcal {H}}\setminus K'| \in \Theta (n/\log n)$. This is also why they achieved only almost everywhere agreement rather than everywhere agreement.

Boyle et al. [22, 23] worked directly with the tree of [11] and came up with a new primitive that suffices to make sure that the adversary can neither prevent an honest party from learning the right output or cause it to accept a wrong value. This new primitive is called *succinctly reconstructed distributed signatures* (SRDS), and allows the parties to aggregate cryptographic signatures along a tree. SRDS have a “robust threshold” property: if enough (say 2/3+ϵ) of the nodes are honest and agree to sign, then even if some internal nodes of the tree are malicious, these malicious nodes cannot prevent a signature from being generated. On the other hand, if few enough (say 1/3-ϵ) nodes agree to sign, then the malicious nodes cannot produce a signature.

The authors of [22, 23] give two instantiations of SRDS. The first is using a PKI and a strong form of (recursively composable) succinct non-interactive arguments of knowledge (SNARKs). Specifically, since the communication tree is of logarithmic depth they eventually need to perform recursive composition log-many times. Thus, to bound the complexity of the knowledge extractor by a fixed polynomial, they need to assume that a single extraction has only constant multiplicative overhead. The second instantiation only requires one-way functions as a cryptographic assumption, but works in the much stronger and less standard *centrally-generated PKI model*, where it is assumed that a central, trusted party generates *all* key pairs. Crucially, such a setup essentially allows to secretly choose and identify a random committee, almost solving the problem we want to solve in the first place.

**(Multi-hop) BARGs to the rescue?** We want to work in the standard PKI model, and to also eliminate the need for SNARKs. A natural attempt is to try to use the exciting recent line of work on BARGs. Recall that a non-interactive batch arguments (BARG) [26, 27], given an NP-language, is an argument system that allows an efficient prover to compute a non-interactive and publicly verifiable “batch proof” of the conjunction of *k* NP instances, with size much smaller than the combined witness length. If any of the *k* instances is false, then no polynomial-time cheating prover must be able to produce an accepting proof. The multi-hop variant [9] allows to compose proofs, enabling mutually distrustful parties to perform distributed computations in a verifiable manner. See Sect. 3.2.2 for the precise definition. BARGs are nowadays known from multiple assumptions like LWE [26, 28], bilinear maps [29–31], decisional Diffie-Hellman (DDH) and quadratic residuosity [27, 32], or obfuscation [33], and the multi-hop variant in particular is known from LWE [9, 10].

Unfortunately, the specific structure of BARGs makes it clear that naively replacing SNARKs with (multi-hop) BARGs in the protocol template of [22, 23] does not work. At a high level, [22, 23]’s certification mechanism consists of a tree of SNARKs, where each non-leaf SNARK has a “running count” of how many valid signatures are present in the corresponding subtree. So a particular node *n* will have left and right children $(c_l, \pi _l)$ and $(c_r, \pi _r)$, where $c_l$ and $c_r$ are counts of the signatures, and $\pi _l$ and $\pi _r$ are SNARKs which attest to those signatures (or are signatures themselves, if *l* and *r* are leaf nodes). Upon seeing these values, node *n* will set $c_n = c_l + c_r$, and generate $\pi _n$ that attests that it saw proofs for its child nodes, *and that they sum up to* $c_n$ . Multi-hop BARGs also allow aggregation of proofs in a tree, but they do not allow this summing-of-counts logic in the internal nodes. With BARGs, the leaves of the tree correspond to NP statements, and the only allowed logic is “all-or-nothing”: either all statements are true, and the root-node proof can be generated attesting to this, or if any statement is not true, then the aggregation fails.

*Our approach*: To alleviate this problem, we completely deviate from the above template and construct a new communication tree which still satisfies the above properties for $K'\subset {\mathcal {H}}$ but we additionally have the following important guarantee: *All internal (non-leaf) nodes have a majority of parties from* ${\mathcal {H}}$ .

**Why does this suffice?** Each internal node in the tree will collect signatures on the candidate string from its members. Because each internal node has an honest majority from $K^{\prime }$, we are guaranteed that the right candidate string will form a majority. Now (because all internal nodes are “honest virtual nodes”), we can use multi-hop BARGs to aggregate the validity of the signatures along the tree. Once all lower level nodes hold an aggregate signature they can send it to their associate parties. The latter are then convinced that this value was held by more than 1/2 of the parties and so it must be correct.

**How do we achieve it?** One can utilize the communication tree of [11] in the following way: ask the members of the supreme committee to jointly sample a *succinct representation* of a new random tree. Say, this can be done by sampling (at the root node) a key for a pseudo-random function (PRF), disseminating it to everyone (along the tree), and locally expanding it to get a new (pseudo-random) new tree. Intuitively, this should satisfy the property that all internal nodes have an honest majority. To see why, recall that at most a 1/3-ϵ fraction of the parties are corrupted. If the members of each internal node were a *freshly sampled* random subset of parties (of poly-logarithmic size), then, by a Chernoff bound, the fraction of corrupted parties in that node would be close to the global fraction, and in particular below 1/2, except with negligible probability. Taking a union bound over the (polynomially many) internal nodes, *all* of them would have an honest majority with all but negligible probability. So obtaining a random tree from a freshly sampled seed suffices.

The above idea almost works. The only problem is that there might exist some “bad” PRF key, which we call a *terrible* key, where the whole tree generated by this key consists of dishonest-majority nodes. To see why a terrible key is a problem, recall how our aggregation works: each internal node holds a statement asserting that a majority of its members signed the candidate seed, and the BARG aggregates these statements along the tree. Soundness of the BARG only guarantees that the aggregate proof cannot be forged *as long as at least one node along the way has an honest majority* (so that the adversary cannot produce a valid witness for that node). If, however, a terrible key exists, then *every* node is dishonest-majority, so the adversary can choose this key and, generate a BARG-based signature which is signed by all nodes in the tree (since they are all majority-dishonest), and present this to any of the honest parties in ${\mathcal {H}}\setminus K'$. Concretely, the corrupt majority in each node can honestly generate a valid witness for that node, and hence a valid aggregate proof for a *wrong* seed. Any such party in ${\mathcal {H}}{\setminus } K'$ will have no way of distinguishing this malicious signature from the honest signature of the true agreed-upon value (recall that a party in ${\mathcal {H}}\setminus K'$ does not necessarily know the true seed). Thus, this scenario allows the adversary to prevent full agreement.

We need some way to prevent generating such a “terrible” tree, i.e., we need to guarantee that, in every possible choice of a tree (equivalently, of a seed), there must be at least one node that has an honest majority. We achieve this property by using a particular method for getting the tree. Instead of choosing it (pseudo-) randomly, we choose only one committee at random. We then “shift” this committee’s members in a particular way so that we are guaranteed that (1) w.h.p all committees consist of an honest majority and (2) for *every* choice of the initial committee, there must be at least one committee that has an honest majority. The second property is what rules out terrible trees, and it holds *deterministically* (for every seed) rather than only with high probability. The reason is a simple counting (pigeonhole) argument: concretely, we sample a single committee of size *d* and define the *n* committees to be its *n* cyclic shifts. In this construction every party appears in exactly *d* committees, so the total number of honest “slots” across all *n* committees is exactly $d\cdot |{\mathcal {H}}|$. By averaging, some committee must contain at least $d\cdot |{\mathcal {H}}|/n$ honest members; when $|{\mathcal {H}}|>n/2$ this is a strict majority. Thus no choice of seed can make *all* committees dishonest-majority. See details in Sect. 4.

The idea of using shifts is somewhat similar to a recent idea due to [19], however, the setting is a bit different and we end up needing new properties. Specifically, in [19], the idea of using shifts was applied to a seed that was sampled differently, and further they use this idea for a different purpose than to generate a tree. In particular, the “no terrible tree” analysis, and in general the entire portion of our work which concerns the communication tree, is novel to this work.

## Model and preliminaries

*Notation*

For any distribution ${\mathcal {X}}$, we denote by $x \leftarrow {\mathcal {X}}$ the process of sampling a value *x* from the distribution ${\mathcal {X}}$. For a set *X* we denote by x←X the process of sampling *x* from the uniform distribution over *X*. For an integer $n \in \mathbb {N}$ we denote by [*n*] the set $\{1,..,n\}$. A function $\textsf{negl}: \mathbb {N} \rightarrow \mathbb {R}$ is negligible if for every constant c>0 there exists an integer $N_c$ such that $\textsf{negl}(\lambda )<\lambda ^{-c}$ for all $\lambda >N_c$. Throughout this paper, honest parties are modeled as uniform probabilistic polynomial-time (PPT) machines, whereas the adversary (as well as the security reductions and extractors we build) is allowed to be non-uniform PPT. We will use λ to denote the computational security parameter. We will use $\textsf{PPT}$ as an acronym for “probabilistic polynomial-time”. We write $1\!\!1_{P}$ for the indicator of a predicate *P*, i.e., $1\!\!1_{P}=1$ if *P* holds and $1\!\!1_{P}=0$ otherwise.

*Model*

We consider *n* parties in a fully connected network (i.e., clique). Each party has a unique ID and the IDs are common knowledge. We assume the IDs are 1,…,n. Parties are uniform and efficient algorithms, i.e., they can be represented as Turing machines that run in polynomial time in the (common) security parameter given in unary representation. Parties can perform arbitrary computation and they have a (private) source of randomness. Communication is private and authenticated, that is, whenever a party sends a message directly to another, the identity of the sender is known to the recipient and moreover the content of the message is hidden from the adversary.^5^ We assume synchronous communication. That is, communication proceeds in rounds; messages are all sent out at the same time at the start of the round, and then received at the same time at the end of the same round.

We assume that there is a $\textsf{PPT}$ adversary that controls up to *t* parties and whose goal is to cause the protocol to fail in some way, depending on the context. The adversary is allowed to be *non-uniform* $\textsf{PPT}$ (while the honest parties need only be uniform). The adversary chooses which parties to control non-adaptively, i.e., it chooses the set of corrupted parties at the start of the protocol. The adversary is malicious: corrupted parties can engage in any kind of deviations from the protocol and send arbitrary messages in the name of the corrupted parties. We emphasize that corrupted parties can send arbitrarily long messages to arbitrary other parties. We assume that the adversary is rushing, that is, it can view all messages sent by the honest parties in a round before the corrupted parties send their messages in the same round.

We further consider protocols in the PKI model, where each party i∈[n] samples for itself a secret key $\textsf{sk}_i$ and a public verification key $\textsf{vk}_i$ from some distribution. The adversary can replace the $\textsf{vk}$’s of corrupted parties arbitrarily and even send inconsistent public keys to different parties. We further assume a common reference string (CRS) which is sampled once at the beginning of time by a trusted party (alternatively, one can think of the CRS as being non-uniformly hardwired into the protocol’s description). The CRS is known to all parties as well as to the adversary. We emphasize that the adversary chooses which parties to corrupt after seeing the CRS and after seeing all (honest) $\textsf{vk}$s, but before the protocol’s execution begins.

In this paper, we say that a protocol is secure if a computationally-bounded adversary cannot contradict any of its properties except with negligible probability. By this, we mean that the probability of the adversary succeeding should be bounded by $\textsf{negl}(n) + \textsf{negl}(\lambda )$, where *n* is the number of parties, and λ is a security parameter which is used to instantiate the cryptographic primitives used in the protocol. For this to be meaningful, we can either assume that λ and *n* are polynomially related (i.e., *n* is upper-bounded by an unspecified polynomial p(·) in terms of λ), and rely on polynomial-hardness assumptions, or we can rely on sub-exponential hardness assumptions and relax this relation, saying that *n* can go up to $2^{\lambda ^{\Theta (1)}}$. We stress that even in the polynomial-hardness regime, none of the constructions in this paper require knowing or specifying the bounding polynomial *p* upfront in order to work.

### Definition of byzantine agreement and friends

*Byzantine agreement*: In this problem, there are *n* parties, $P_1,\ldots ,P_n$. At most *t* of the parties may be corrupted. Each party $P_i$ begins with an input bit $x_i \in \{0,1\}$ and outputs a bit $y_i$. The goal of the protocol is (with high probability) for all honest parties to terminate and, upon termination, agree on a bit held by at least one honest party at the start. This should hold even when the *t* corrupted parties collude and actively try to prevent it. More precisely, the Byzantine agreement problem is defined as a protocol that satisfies with high probability the following *agreement*, *validity*, and *termination* properties:

- *Agreement*: For every pair of honest parties $P_i$ and $P_j$ it holds that $y_i = y_j$.
- *Validity*: If there exists a bit *x* such that for every honest party $P_i$ it holds that $x_i = x$, then the common output is *x*.
- *Termination*: Every honest party eventually outputs a bit.

*Committee election and broadcast*: We now state the analogous definitions for broadcast, committee election, and leader election. As before, all properties are only required to hold with high probability, and only for honest parties.

In **broadcast**, there is a designated sender *S* who holds an input message *m*, and each party outputs a message. The following are required:

- *Agreement*: All honest parties output the same message.
- *Validity*: If the sender *S* is honest, then all honest parties output *m*.
- *Termination*: Every honest party eventually outputs a message.

In **committee election**, the parties agree on a subset (committee) C⊆[n] of a prescribed size *s*. Denoting by *B* the set of corrupted parties, the following are required:

- *Agreement*: All honest parties output the same committee *C*.
- *Quality*: The fraction of corrupted parties in the elected committee is close to their global fraction; that is, $|C\cap B|/|C| \le |B|/n + o(1)$.
- *Termination*: Every honest party eventually outputs a committee.

Here we think of the committee size *s* as small (e.g., poly-logarithmic in *n*), so that a “good” committee can subsequently run a heavier protocol among its members.

In **leader election**, the parties agree on a single party (the leader), i.e., committee election with s=1, and the requirements are:

- *Agreement*: All honest parties output the same leader.
- *Validity*: The elected leader is honest with at least some constant probability (over the randomness of the protocol).
- *Termination*: Every honest party eventually outputs a leader.

#### Quorum to byzantine agreement, broadcast, and committee election

It is not at all obvious if one can translate a protocol for one of the above tasks into another one *while preserving scalability*. One naive idea is to choose a small committee and let it do most of the work. That is, elect a small committee and run a “heavy-weight” protocol among the committee members for task X, where X could be (for instance) leader election, Byzantine agreement, or broadcast. If the committee is sufficiently small, then we can afford to execute rather inefficient protocols among the committee members. The problem with this approach is that it results with highly unbalanced protocols: members would need to communicate with all other parties.

There is a useful abstraction called a **quorum** that does imply all of the above applications as a special case. A quorum is a collection of *n* committees where (1) each committee contains roughly the same fraction of bad parties as in the total population, (2) Each party is a member in roughly the same number of committees. Once we have a quorum, it is relatively easy to obtain all other above-mentioned abstractions with essentially no extra complexity (as noted in [11]). For completeness, we sketch the details next.

The following lemma formalizes this reduction; we give the intuition first and then a concise proof.

Consider the Byzantine agreement problem first. Given a quorum, each party sends its bit to its associated committee members. The committees now perform agreement in a tree-like fashion by say computing the majority value of their input bits. Lastly, all committees distribute the agreed upon bit to their respective associated parties. Broadcast is implemented in a similar fashion by performing broadcast in a tree-like fashion between the committees defined via the quorum (again, since these committees are behaving honestly, this is easy). Committee election is a special case of quorum (the first committee in the quorum is a good committee). Leader election can be implemented by running a naive (heavy-weight) protocol among a chosen committee.

We formalize the above as follows. Below, by a *good quorum* we mean a collection of *n* committees, each of poly-logarithmic size, in which every committee has an honest majority and every party belongs to only poly-logarithmically many committees (this is exactly the notion made precise in Definition 2 and Sect. 4).

##### Lemma 1

(Quorum to agreement tasks) Suppose all honest parties hold (agree on) a good quorum of *n* committees, each of size $d\in {\tilde{O}}(1)$, such that every committee has an honest majority and every party belongs to at most ${\tilde{O}}(1)$ committees. Then there are protocols for Byzantine agreement, broadcast, and committee or leader election that terminate within ${\tilde{O}}(1)$ rounds in which each party sends $\textsf{poly}(\lambda ,\log n)$ bits overall.

##### Proof sketch

Fix a good quorum and associate its *n* committees with the nodes of a fixed complete binary tree of depth O(logn) (say, committee $C_i$ with the *i*-th node). Since every committee has an honest majority, each committee can reliably compute a majority function of its inputs and reliably relay messages to neighboring committees (a corrupted minority cannot change the majority value).

For *Byzantine agreement*, each party $P_i$ sends its input bit to the members of its associated committee; committees then aggregate up the tree by (honest-majority) majority vote, the root committee fixes the agreed bit, and the value is propagated back down the tree and finally to the associated parties. Agreement and validity follow because every committee along every path faithfully computes and forwards the majority, and termination follows since the tree has depth O(logn). *Broadcast* is identical except that the sender first sends its message to its committee (validity for an honest sender follows as its message reaches the root unaltered). *Committee election* is immediate, as (say) the first committee of the quorum is itself a good committee of the required quality. *Leader election* is obtained by electing a good committee as above and then running a heavy-weight (polynomial) leader-election protocol among its ${\tilde{O}}(1)$ members.

For the complexity, each committee has $d\in {\tilde{O}}(1)$ members, each party belongs to ${\tilde{O}}(1)$ committees, the tree has depth $O(\log n)\subseteq {\tilde{O}}(1)$, and each message is a bit or a $\textsf{poly}(\lambda ,\log n)$-size object; hence each party sends $\textsf{poly}(\lambda ,\log n)$ bits over ${\tilde{O}}(1)$ rounds. □

### Cryptographic building blocks

*Learning with Errors*: The learning with errors ($\textsf{LWE}$) problem, introduced by Regev [38] and denoted $\textsf{LWE}_{n,m,q,\chi }$ for parameters $n,m,q \in \mathbb {N}$ and for a distribution χ supported over $\mathbb {Z}$, is to distinguish between the following pair of distributions

$$\begin{aligned} (\textbf{A},\vec {s} \textbf{A} + \vec {e} \bmod q) \quad \text {and}\quad (\textbf{A},\vec {u}), \end{aligned}$$

where $\textbf{A}\leftarrow \mathbb {Z}^{n\times m}_{q}$, $\vec {s} \leftarrow \mathbb {Z}^{1\times n}_{q},\ \vec {e} \leftarrow \chi ^{1\times m}$, and $\vec {u} \leftarrow \mathbb {Z}^{1\times m}_{q}$. The $\textsf{LWE}_{n,m,q,\chi }$ assumption states that the two distributions are computationally indistinguishable. Informally, it asks to tell noisy linear equations over $\mathbb {Z}_q$ apart from uniformly random ones.

In this paper, we rely on multi-hop BARGs for NP (to be defined in Sect. 3.2.2, below) in a generic manner, that in turn, can be constructed based on $\textsf{LWE}$. Since we rely on a standard polynomially-hard version of BARGs, we need a standard version of LWE with polynomial ratio between modulus and noise.

#### Digital signatures

A digital signature allows one party to “convince” another party that a third party indeed “approved” a given message. The notion that suffices for this paper is that of *existential unforgeability*, where it is required to be computationally infeasible to generate a valid message-signature pair even after seeing as many such pairs for different messages as needed. We first describe the syntax and then formalize the security notion via a game between a challenger and an adversary.

A signature scheme consists of three polynomial-time procedures ${\mathcal {S}} = (\textsf{Gen}, \textsf{Sign}, \textsf{Verify})$ with the following syntax:

- $\textsf{Gen}(1^\lambda ) \rightarrow \textsf{vk}, \textsf{sk}$: given security parameter $1^\lambda$, the procedure outputs a verification key $\textsf{vk}$ and signing key $\textsf{sk}$ pair.
- $\textsf{Sign}(\textsf{sk}, m) \rightarrow \sigma$: given signing key $\textsf{sk}$ and a message *m*, the procedure outputs a signature σ.
- $\textsf{Verify}(\textsf{vk}, m, \sigma ) \rightarrow \{0, 1\}$: given verification key $\textsf{vk}$, message *m*, and a signature σ, the procedure outputs 0 (for “fail”) or 1 (for “success”).

*Correctness*: A signature scheme should satisfy that honestly generated signatures verification always succeed. That is, for all $\lambda \in {\mathbb {N}}$, $\textsf{vk}, \textsf{sk}\leftarrow \textsf{Gen}(1^\lambda )$, and $m \in \{0,1\}^*$, it holds that

$$\begin{aligned} \Pr [\textsf{Verify}(\textsf{vk}, m, \textsf{Sign}(\textsf{sk}, m))=1] = 1. \end{aligned}$$

*PKI existential unforgeability*:

We require that for any $\textsf{PPT}$ adversary ${\mathcal {A}}$, there is a negligible function $\textsf{negl}(\cdot )$ such that the probability to win the unforgeability game is at most $\textsf{negl}(\lambda )$. That is,

$$\begin{aligned} \Pr [\textsf{ExUnfg}_{{\mathcal {S}},{\mathcal {A}}}(\lambda ) = 1 ] \le \textsf{negl}(\lambda ), \end{aligned}$$

where the game $\textsf{ExUnfg}_{{\mathcal {S}}, {\mathcal {A}}}(\lambda )$ is defined as:

1. ${\mathcal {A}}(1^\lambda )$ specifies an integer *k* by writing it in unary.
2. Initialize ${\mathcal {M}} = \emptyset$ and run $\{(\textsf{vk}_i, \textsf{sk}_i)\leftarrow \textsf{Gen}(1^\lambda )\}_{i \in [k]}$. Send $\{\textsf{vk}_i\}_{i \in [k]}$ to ${\mathcal {A}}$.
3. ${\mathcal {A}}$ chooses a set ${\mathcal {C}}\subseteq [k]$ and gets back $\{\textsf{sk}_i\}_{i \in {\mathcal {C}}}$.
4. ${\mathcal {A}}$ gets access to a signing oracle that gets as input pairs of the form (*i*, *m*) for $i\in [k]\setminus {\mathcal {C}}$ and returns $\sigma _{i,m} = \textsf{Sign}(\textsf{sk}_i, m)$. ${\mathcal {A}}$ makes as many queries as it wants (adaptively), and upon every such query, the experiment updates ${\mathcal {M}} = {\mathcal {M}} \cup \{(i,m)\}$. When ${\mathcal {A}}$ queries $(\bot ,\bot )$, the experiment proceeds to the next step.
5. ${\mathcal {A}}$ outputs $(i^*, m^*, \sigma ^*)$ and the experiment outputs 1 (i.e., adversary wins) if (1) $i^*$ is not among the set of corrupted keys ${\mathcal {C}}$, (2) $(i^*,m^*)$ does not belong to the list of queries ${\mathcal {M}}$, and (3) the signature $\sigma ^*$ is a valid signature of $m^*$ with respect to $\textsf{vk}_{i^*}$. That is, $i^* \in [k] \setminus {\mathcal {C}}\;\wedge \; (i^*,m^*) \notin {\mathcal {M}} \;\wedge \; \textsf{Verify}(\textsf{vk}_{i^*}, m^*, \sigma ^*)$.

##### Remark 1

(Equivalence to standard existential unforgeability) The standard definition of existential unforgeability of signatures is a special case of the above where k=1 and ${\mathcal {C}}= \emptyset$. By a standard “guessing” argument the two notions are existentially equivalent. That is, to translate an attacker of the above definition to an attacker in the “single-key” variant, we can embed the challenge $\textsf{vk}$ into the list of $\textsf{vk}$ at a random index. If this index ends up being corrupted (i.e., in ${\mathcal {C}}$), the game aborts. Otherwise, the game proceeds normally. A successful attack to the PKI existential unforgeability game will translate into a successful attack in the single-key existential unforgeability game with probability smaller by 1/*n* factor.

The above definition of digital signatures is standard and satisfied by many known constructions. For instance, the one-way function-based construction satisfies it [34, 35] (which then implies an LWE-based construction, directly).

#### Multi-Hop BARGs for NP

Let $R(\cdot , \cdot )$ be an NP relation, and let $x_1, \dots , x_n$ be *n* public statements. A BARG scheme stands for publicly verifiable non-interactive argument system for batch-NP. Such a scheme allows a prover to prove that it knows *n* witnesses such that for all $i \in [n], R(x_i, w_i) = 1$, using total communication *o*(*n*). A multi-hop BARG scheme extends this notion to a distribute system where there are *n* provers, each one holding a different witness. A multi-hop BARG allows the provers to jointly and aggregate a proof that they know *n* witnesses using *o*(*n*) communication for each prover. In two concurrent recent works [9, 10], a multi-hop BARG was given. We give the definition of multi-hop BARG from [9], except that we simplify it slightly by fixing the aggregation pattern to be in a full binary tree-like manner.

##### Definition 1

*(Multi-hop BARG scheme with fixed aggregation pattern)* Let $R(\cdot , \cdot )$ be a NP-relation and let $\lambda ,n\in {\mathbb {N}}$ be two parameters. Let $X = (x_1, \dots , x_n)$ be *n* public statements, and let the aggregation pattern be the full binary tree with *n* labeled nodes (denoted *T*). A multi-hop BARG scheme with fixed aggregation pattern for *R* consists of the following (randomized) polynomial time algorithms:

- $\textsf{Gen}(1^\lambda , i^*) \rightarrow \textsf{crs}, \textsf{td}$. The setup algorithm receives as input the security parameter λ, and an index of statement $i^*$. It returns the $\textsf{crs}$ (common reference string) and trapdoor $\textsf{td}$.
- ${\mathcal {P}}(\textsf{crs}, x, w) \rightarrow \pi /\bot$. The proof generation algorithm receives the common reference string $\textsf{crs}$, an instance *x*, and a potential witness *w*. If R(x,w)=1, it outputs a proof π; otherwise, it returns ⊥.
- $\textsf{AggProve}(\textsf{crs}, X, i, \pi _i, \pi _L, \pi _R) \rightarrow \pi$. The aggregation algorithm receives as input the *n* instances *X*, an index *i* in the aggregation tree, the proof $\pi _i$ associated with the *i*th instance $x_i$, and the aggregated proofs $\pi _L$ and $\pi _R$ of the left and right subtrees of index *i*, respectively. It returns an aggregated proof π associated with the subtree of index *i*.
- ${\mathcal {V}}(\textsf{crs}, X, i, \pi ) \rightarrow \{0,1\}$. The verification algorithm gets as input the *n* instances *X*, an index *i*, and a proof π. It returns 1 if π is valid proof for the aggregated statements with respect to the subtree; otherwise, it returns 0.

The scheme need to satisfy the following:

- *Efficiency*: The size of $\textsf{crs}, \textsf{td}$ is at most $\textsf{poly}(\lambda ,\log n)$, and the size of a (combined) proof corresponding to *T* is at most $\max _{i \in [n]}|w_i| + \textsf{poly}(\lambda ,\log n)$.
- *Correctness*: For any $\textsf{crs}, \textsf{td} \leftarrow \textsf{Gen}(1^\lambda ,i^*)$, and for any *X* and valid witnesses:
  - For every leaf *j* of *T*:
    $$\begin{aligned} {\mathcal {V}}(\textsf{crs}, X, j, {\mathcal {P}}(\textsf{crs}, x_j, w_j)) = 1. \end{aligned}$$
  - For every internal node k∈[n] the following holds. Let $k_L$ and $k_R$ be *k*’s left and right children, respectively. Assume that $\pi _L$ and $\pi _R$ are proofs associated with $k_L$ and $k_R$ such that ${\mathcal {V}}(\textsf{crs}, X, k_L, \pi _L) = 1$ and ${\mathcal {V}}(\textsf{crs}, X, k_R, \pi _R) = 1$. Then,
    $$\begin{aligned}&{\mathcal {V}}(\textsf{crs}, X, k, \textsf{AggProve}\\&\quad \times (\textsf{crs}, X, k, {\mathcal {P}}(\textsf{crs}, x_k, w_k), \pi _L, \pi _R)) = 1. \end{aligned}$$
- *Index hiding*: For any $\textsf{PPT}$ adversary ${\mathcal {A}}$, and any i,j∈[n]:
  $$\begin{aligned}&\left| \Pr _{\textsf{crs}\leftarrow \textsf{Gen}(1^\lambda , i)}[{\mathcal {A}}(\textsf{crs}, i, j) = i] \right. \\&\qquad \left. - \Pr _{\textsf{crs}\leftarrow \textsf{Gen}(1^\lambda , j)}[{\mathcal {A}}(\textsf{crs}, i, j) = i]\right| \le \textsf{negl}(\lambda ). \end{aligned}$$
- *Somewhere argument of knowledge*: There exists a $\textsf{PPT}$ extractor ${\mathcal {E}}$, such that for any $\textsf{PPT}$ adversary ${\mathcal {A}}$:

##### Theorem 2

(Theorem 1.3 in [9]) Assume hardness of $\textsf{LWE}$ against non-uniform polynomial-time attackers. A multi-hop BARG for all NP satisfying Definition 1 exists.

Above, we assume that *n*, the number of instances in the batch, and λ, the computational security parameter, are polynomially related. One can support even *n* that grows sub-exponentially with λ (or, alternatively, λ that is only poly-logarithmic in *n*) if we assume sub-exponential hardness of $\textsf{LWE}$.

Our agreement protocol is eventually proven secure by a reduction to the security of the multi-hop BARG and an underlying signature scheme. We have stated the existence of multi-hop BARGs from $\textsf{LWE}$ because this is the way that [9, 10] state it. However, the latter works actually show a generic construction of multi-hop BARGs from two primitives (1) somewhere-extractable hash functions (SEH) [26, 36], and (2) standard batch arguments (i.e., not necessarily multi-hop). These were first constructed from $\textsf{LWE}$ [26, 36], and more recently using other assumptions (e.g., $\textsf{DLIN}$ or subexponentially-hard $\textsf{DDH}$) [31]. By relying on these results (and noting that signatures exist too under these assumptions), we can get our results also from those assumptions. Lastly, we mention that our protocols only require multi-hop BARG that supports logarithmically-many hopes.

## Quorum agreement protocol

In this section we provide a scalable protocol used to agree on a “good” quorum. Recall that a quorum is a collection of *n* committees $C_1,\ldots ,C_n\in [n]^d$ (i.e., size *d* subsets of parties). In our context, each committee will be of size $d=\log ^2n$ and a quorum is good if the following conditions hold. Denote by *B* the set of parties the adversary controls, and recall that $|B|\le (1/3-\epsilon )n$ for ϵ>0.

1. For each i∈[n], $|C_i \setminus B| > d/2$. That is, there is an honest majority in each committee.
2. For each j∈[n], $\sum _{i\in [n]} |C_i \cap \{j\}| \in {\Theta (d)}$. That is, each party appears in about *d* committees (in fact, our construction places every party in exactly *d* committees).

The protocol that we describe in this section results with a good quorum as described above, and does so using only $\textsf{poly}(\lambda ,\log n)$ per-party communication and ${\tilde{O}}(1)$ rounds. All of our protocols succeed with very high probability, i.e., all but negligible probability of error in the security parameter and in *n*.

### Theorem 3

Assume a PKI, a CRS, and the hardness of LWE. Then, there is a protocol for generating a good quorum (with probability at least $1 - \textsf{negl}(n)$), secure against a $\textsf{PPT}$ adversary that statically corrupts up to 1/3-ϵ fraction of parties for any constant ϵ>0. Furthermore, the protocol terminates within ${\tilde{O}}(1)$ rounds and each party sends $\textsf{poly}(\lambda ,\log n)$ bits overall.

As immediate corollaries, we obtain protocols for classical agreement tasks such as byzantine agreement, broadcast, and committee or leader election. The construction that achieves this transformation is immediate and is described in Sect. 3.1.1.

### Theorem 4

(Restatement of Theorem 1) Assume a PKI, a CRS, and the hardness of LWE. Then, there are protocols for byzantine agreement, broadcast, and committee or leader election, secure against a $\textsf{PPT}$ adversary that statically corrupts up to 1/3-ϵ fraction of parties for any constant ϵ>0. Furthermore, these protocols terminate within ${\tilde{O}}(1)$ rounds and each party sends $\textsf{poly}(\lambda ,\log n)$ bits overall.

*Section organization*: In Sect. 4.1 we present a protocol that makes almost all parties agree on a random string. That is, at the end of this phase, each party *i* holds a string $\textsf{seed}_i$. For all but *o*(1) fraction of parties, there is a string $\textsf{seed}$ such that all $\textsf{seed}_i$’s are equal to it. The *o*(*n*) parties that do not hold $\textsf{seed}$ are called unknowledgeable. There is no guarantee on the $\textsf{seed}_i$ of the unknowledgeable parties. Furthermore, $\textsf{seed}$ is distributed uniformly from $\{0,1\}^{\log ^3 n}$. It is not guaranteed that a party knows whether it is knowledgeable or not. Then, in Sect. 4.2 we show that the output of the previous protocol can be used to sample from a strong family of good quorums. That is, we construct a family of good quorums with additional properties that are crucial for the next step in our protocol. Lastly, in Sect. 4.3 we preset the full protocol that causes all parties to agree on $\textsf{seed}$ and thereby implies a good quorum.

### Almost everywhere agreement on a seed

Recall that [11]’s protocol establishes a O(logn)-degree communication tree (which is essentially a sparse overlay network) in which each node is assigned with a committee of ${\tilde{O}}(1)$ parties. The guarantees are that the ${\tilde{O}}(1)$-size root committee has a 2/3 honest majority and almost all of the parties are connected to the supreme committee via the communication tree. Denote *root* the root committee.

We note that this building block was recently revisited by Bhangale, Liu-Zhang, Loss, Nayak, and Yandamuri [39], who observed that the analysis of [11] is incomplete: the “silencing” mechanism (used to keep communication low) interacts poorly with sampler-induced bad elections, and the original argument does not adequately bound the number of honest parties that get silenced. They give a modified protocol that restores the intended almost-everywhere guarantees, with poly-logarithmic communication per party, for t<(1/3-ϵ)n. Whenever we invoke almost-everywhere agreement / the communication tree of [11] as a black box, we rely on this corrected construction (rather than on the original analysis alone).

Before describing the protocol, we recall the primitives that it composes. We run a standard MPC protocol among the parties of the root committee in order to sample a fresh random string $\textsf{seed}$ of length $\log ^3 n$. Concretely, we use the BGW protocol [6], which assumes pairwise private channels and a broadcast channel among the participating parties. Private channels are already part of our model (and can be implemented from a PKI), whereas the broadcast channel among the (poly-logarithmically many) root-committee members is implemented using a standard—polynomial but not necessarily scalable—broadcast protocol, such as that of Berman, Garay, and Perry [37]. Since BGW is executed only among the ${\tilde{O}}(1)$ members of the root committee, its polynomial cost translates into ${\tilde{O}}(1)$ cost overall.

$$\underline{{AlmostEverywhereAgreementOnASeed}}$$:

1. All the parties run the protocol of [11] (as corrected by [39]) to generate the communication tree.
2. Each party i∈root samples $\textsf{seed}_i \leftarrow \{0,1\}^{\log ^3 n}$. The parties in the root committee run the BGW MPC protocol [6] to compute $\textsf{seed}{\leftarrow \bigoplus _{i \in root} \textsf{seed}_i}$ (the required broadcast channel is realized via [37], as discussed above).
3. The root committee distributes $\textsf{seed}$ to the leaves using the communication tree from step 1.
4. Each leaf node $N_i$ sends $\textsf{seed}$ to party $P_i$, who stores the value it received most often.

#### Theorem 5

For any $\textsf{PPT}$ adversary ${\mathcal {A}}$ that statically corrupts up to a 1/3-ϵ fraction of parties, for any constant ϵ>0, with probability at least $1 - \textsf{negl}(n) - \textsf{negl}(\lambda )$, at the end of the protocol, a 1-o(1) fraction of honest parties agree on a uniform string $\textsf{seed}\in \{0,1\}^{\log ^3 n}$. In addition, the protocol terminates after ${\tilde{O}}(1)$ rounds, and each party sends $\textsf{poly}(\lambda ,\log n)$ bits overall.

We prove Theorem 5 by direct composition of the results of [6, 11, 37, 39]. We first recall the two building blocks that we compose, and then give the proof.

We use BGW to obtain a coin-flipping protocol. Below we state a weak version of the result of [6] that suffices for us. Here, security means that the protocol computes the prescribed function even against a malicious adversary who controls the corrupted parties; in particular, the adversary is allowed to choose the inputs of the corrupted parties.

#### Theorem 6

([6]) Given private channels between each pair of parties and a broadcast channel, for every *n*-ary polynomially-computable function *f*, and for any (computationalally unbounded) adversary ${\mathcal {A}}$ that statically corrupts at most n/3-1 parties, there exists a protocol that terminates after $\textsf{poly}(n,|f|)$ rounds, and in which each party sends $\textsf{poly}(n, |f|)$ bits. At the end, each party holds $f(x_1, \dots , x_n)$, where $x_1, \dots , x_n$ are the parties^’^ inputs.

In particular, taking *f* to be the exclusive-or (XOR) of one-bit inputs yields a coin-flipping protocol for a single unbiased bit, as long as at least one participating party is honest. Running this bit-by-bit (equivalently, computing a single multi-bit XOR over $\text {GF} (2)$) yields the following corollary.

#### Corollary 1

(Coin-flipping a bitstring) Given private channels between each pair of parties and a broadcast channel, and for any adversary that statically corrupts at most n/3-1 of the participating parties, there is a protocol that terminates after $\textsf{poly}(n,\ell )$ rounds, in which each party sends $\textsf{poly}(n,\ell )$ bits, at the end of which all parties hold the same string $s\in \{0,1\}^{\ell }$. Moreover, if at least one participating party is honest, then *s* is distributed uniformly over $\{0,1\}^{\ell }$.

For the above theorem, recall that our model already assumes private channels, and further we can use a well-known protocol for broadcast [37]. We now state the result of [11] (as corrected by [39]) that gives a communication tree.

#### Theorem 7

([11, 39]) For any (computationally unbounded) adversary ${\mathcal {A}}$ that statically corrupts a 1/3-ϵ fraction of parties, for any constant ϵ>0, there is a ${\tilde{O}}(1)$-round protocol, where each party sends at most ${\tilde{O}}(1)$ bits during the protocol, that creates a communication tree with the following properties:

1. There is *n* leaf nodes, where each internal node has logn sons, so the tree depth is $O(\log n/\log \log n)$.
2. Each leaf node of the tree is assigned a set of $\log ^5 n$ processors. Each processor is assigned to $O(\log ^4 n)$ leaf nodes. All processors know exactly which processors participate in which leaf nodes.
3. Each internal node of the tree is assigned a set of $\log ^3 n$ processors. Each processor is assigned to $O(\log ^4 n)$ internal nodes per level, and it knows the other parties that assigned to that nodes.
4. With probability at least $1 - \textsf{negl}(n)$, at most a $3/ \log ^2 n$ fraction of the nodes in the tree are bad (contains dishonest majority), and all but a 3/logn fraction of the leaf nodes have a good path (all node in the path are good) to the root node.
5. Suppose there is a good path from A to the root, and let B be the parent of A. Then all processors in A know all processors in B, and all processors in B know all processors in A.

Now we are ready to prove the correctness of the combined protocol.

#### Proof of Theorem 5

From Theorem 6 it follows that after Step 2 each party in the root will hold $\textsf{seed}$. Since there exists at least one honest party in the root, the XOR of all these independently chosen values is uniformly random. Also, since there are $\log ^3 n$ parties in the tree root, the end of Step 2 will be reached after ${\tilde{O}}(1)$ rounds and each party will send at most ${\tilde{O}}(1)$ bits, overall. Lastly, since there are only *o*(1) leaves nodes that are “bad” or have “bad” path to the root, at the end of the protocol, 1-o(1) fraction of parties will hold $\textsf{seed}$. □

### Sampling a strong quorum

Recall that a quorum is a collection of *n* committees. Roughly, a quorum is good if each committee has a fraction of corrupt parties similar to the fraction of total corrupt parties (out of *n*). Also, we shall require a balancedness property meaning that each party appears roughly in the same number of committees. These properties have been considered before and are not new to this work. We introduce and require a new property called *no terrible quorums* meaning that in every possible (adversarially chosen) quorum there is at least one “good” committee. Looking ahead, this is precisely the property that prevents the adversary from forging a BARG-based proof for a *wrong* seed: for *any* seed the adversary might try to convince honest parties of, the induced quorum contains at least one committee $C_{i^*}$ with an honest majority. Since the BARG setup embeds a random index $i^*$ that is hidden from the adversary (index hiding), with probability at least 1/*n* this good committee is exactly the one from which the somewhere-extractor extracts a witness. Extracting a valid witness for that committee would require a valid signature on the wrong seed by an honest party, contradicting unforgeability. In other words, “no terrible quorums” combined with somewhere extraction (and a pigeonhole argument over committees) is what rules out proofs for wrong seeds; see Claim 5 and lemma 4.

#### Definition 2

*(Good family of quorums)* A family of quorums is a family ${\mathcal {Q}}= \{{\mathcal {Q}}_n\}_{n\in {\mathbb {N}}}$. For each $n\in {\mathbb {N}}$, each member $Q\in {\mathcal {Q}}_n$ is a set of *n* committees $Q=(C_1,\ldots ,C_n) \in ([n]^d)^n$, where the size of each committee is $d=\log ^2 n$. Note that each $C_i$ is a multi-set. The family ${\mathcal {Q}}$ is said to be good if

1. *Efficient sampling*: There is a deterministic polynomial-time procedure $\textsf{QSample}(1^n,\textsf{seed})$ that gets as input $n\in \mathbb {N}$ and $\textsf{seed}\in \{0,1\}^{\log ^3 n}$, and outputs a quorum $Q\in {\mathcal {Q}}_n$.
2. *Good committees*: For every $n\in {\mathbb {N}}$, every H⊆[n] such that $|H|>n/\log \log n$, with high probability over the choice of the quorum $Q = (C_1,\ldots ,C_n)$, the density of *H*-members in $C_i$ is very close to their density in [*n*]. That is,
3. *Balanced committees*: For every $n\in {\mathbb {N}}$, with high probability over the choice of the quorum $Q = (C_1,\ldots ,C_n)$, every party appears in *O*(*d*) committees overall. That is,
4. *No terrible quorums*: For every $n\in {\mathbb {N}}$ and every H⊆[n], for every possible choice of a quorum $Q = (C_1,\ldots ,C_n)$, there exists at least one committee $C_i$ where the density of *H*-members in $C_i$ is at least |*H*|/*n*. That is, for every $\textsf{seed}\in \{0,1\}^{\log ^3 n}$ such that $(C_1,\ldots ,C_n) = \textsf{QSample}(1^n, \textsf{seed})$, it holds that
  $$\begin{aligned} \exists i\in [n]:\frac{1}{d}\cdot \sum _{z\in C_i} 1\!\!1_{z\in H} \ge \frac{|H|}{n}. \end{aligned}$$

#### Theorem 8

There is an implementation of $\textsf{QSample}$ such that for every $n\in \mathbb {N}$, $\textsf{QSample}(1^n,\cdot )$ results with a good family of quorums as defined in Definition 2.

#### Proof

We define the $\textsf{QSample}$ procedure as follows^6^:

$$\underline{\textsf{QSample}(1^n, \textsf{seed}), \hbox { where }|\textsf{seed}| = \log ^3n}$$:

1. Parse $\textsf{seed}$ as $d \triangleq \log ^2 n$ elements in [*n*], denoted $C^*=P^*_1,\ldots ,P^*_d$.
2. Define $C_i = P^*_1 + i, \ldots P^*_d + i$ (all arithmetic is mod(n) and the *n*th party is identified by 0).
3. Output $$Q = (C_1,\ldots ,C_n)$$.

The efficiency property of this construction is immediate by description. We argue that we get balanced committees for every $\textsf{seed}$ (i.e., with probability 1). Fix party j∈[n] and $\textsf{seed}\in \{0,1\}^{\log ^3n}$. Denote $Q=(C_1,\ldots ,C_n)= \textsf{QSample}(1^n,\textsf{seed})$ and let $C^*$ be as above. It holds that

$$\begin{aligned} \begin{aligned} \sum _{i=1}^n \sum _{y\in C_i} 1\!\!1_{j = y}&= \sum _{i=1}^n \sum _{z \in C^*} 1\!\!1_{j = z + i \bmod n} \\&= \sum _{z \in C^*} \sum _{i=1}^n 1\!\!1_{j = z + i \bmod n} = \sum _{z \in C^*} 1 = |C^*| = d. \end{aligned} \end{aligned}$$

The fact that the resulting committees are good (with high probability) follows by an application of Chernoff’s bound along with a union bound. Specifically, fix i∈[n] and abbreviate the event of interest by

$$ \textsf{Good}_i \;\triangleq \; \left[ \frac{1}{d}\sum _{z\in C_i} 1\!\!1_{z\in H} \;\ge \; \left( 1- \frac{1}{\log \log n}\right) \frac{|H|}{n}\right] . $$

By Chernoff’s bound (recall $d=\log ^2 n$),

$$\begin{aligned} \Pr _{\begin{array}{c} \textsf{seed}\leftarrow \{0,1\}^{\log ^3 n},\\ (C_1,\ldots ,C_n) = \textsf{QSample}(1^n, \textsf{seed}) \end{array}}\!\left[ \textsf{Good}_i\right] \;&\ge \; 1-n^{-\frac{\log n \cdot |H|}{2n \cdot \log \log ^2 n}}\\ \;&\ge \; 1-\textsf{negl}(n), \end{aligned}$$

where the last inequality follows since $|H|>n/\log \log n$. The second property of a good quorum now follows by a union bound over all i∈[n].

For the no terrible quorum property, observe that each party appear in exactly *d* committees and therefore the total number of honest parties in all quorums together is d·|H|. By the pigeonhole principle, there must be one $C_i$ with (d·|H|)/n parties from *H*. □

### Everywhere agreement on a quorum

In this section we explain the final protocol, resulting with a good quorum known to all honest parties. At a very high level, we utilize the almost everywhere protocol from Sect. 4.1 to get a seed that is known to almost everyone. Then, we let each party expand its candidate seed to a quorum (that is agreed upon by almost everyone) using Theorem 8. We then use the multi-hop BARG to aggregate signatures along a tree of the quorum committees. Once the committees get an aggregated signature of all other committees they can pass on the candidate string to their associated parties.

*Building blocks*: Our protocol utilizes the following primitives:

1. A signature scheme ${\mathcal {S}} = (\textsf{Gen}, \textsf{Sign}, \textsf{Verify})$, as in Sect. 3.2.1.
2. A multi-hop BARG for NP $\textsf{BARG} = (\textsf{Gen}, {\mathcal {P}},\textsf{AggProve}, {\mathcal {V}})$, as in Definition 1. The NP relation that we use is defined next.

*Committee signature relation*: Recall that *d* is the size of a committee in the quorum. The NP relation we utilize in our BARGs is defined next.

#### Definition 3

*(NP relation for BARGs)* We define the following NP-relation:

$$\begin{aligned}&R_{{\mathcal {S}}, f, \vec {\textsf{vk}}, \textsf{seed}}(i, \{\sigma _j\}_{j \in [d]}) \\&\quad = {\left\{ \begin{array}{ll} 1 &  i \in [n] \wedge \sum _{j \in [d]} {\mathcal {S}}.\textsf{Verify}(\textsf{vk}_{f(i, \textsf{seed}, j)}, \textsf{seed}, \sigma _j) > d / 2,\\ 0 &  otherwise, \end{array}\right. } \end{aligned}$$

where $f :[n] \times \{0,1\}^* \times [d] \rightarrow [n]$ is a fixed polynomial-time computable function, $\vec {\textsf{vk}}$ is a vector of fixed public verification keys, and $\textsf{seed}$ is a fixed seed string.

Above, we view $f(i, \textsf{seed}, \star )$ as a function that outputs a multi-set of *d* parties (i.e., a committee). So, the above relation classifies a valid witness for index *i* as one containing a majority of valid signatures on $\textsf{seed}$ from the multi-set members.

*The QuorumAgree protocol*: We now present the QuorumAgree protocol. Let *T* be an abstract binary tree with *n* nodes. Each of the nodes is virtually associated with a committee (in some arbitrary but fixed manner). Let *Depth*(*T*) be *T*’s depth, i.e., the number of nodes in the longest path from the root to the leaves (so roughly Depth(T)=logn). For a node *j* in the tree, let *Parent*(*j*) be its parent, *Left*(*j*) and *Right*(*j*) be its left and right children, respectively. Throughout the protocol description below, when we say that a committee does something (e.g., sends a message or computes a value), we mean that every party in the committee performs this operation and labels it with its identity and its view of the committee index. Importantly, since every party holds its own candidate for $\textsf{seed}$, the protocol may proceed differently for different parties. We write ← for assignment and = for equality.

**Figure:**
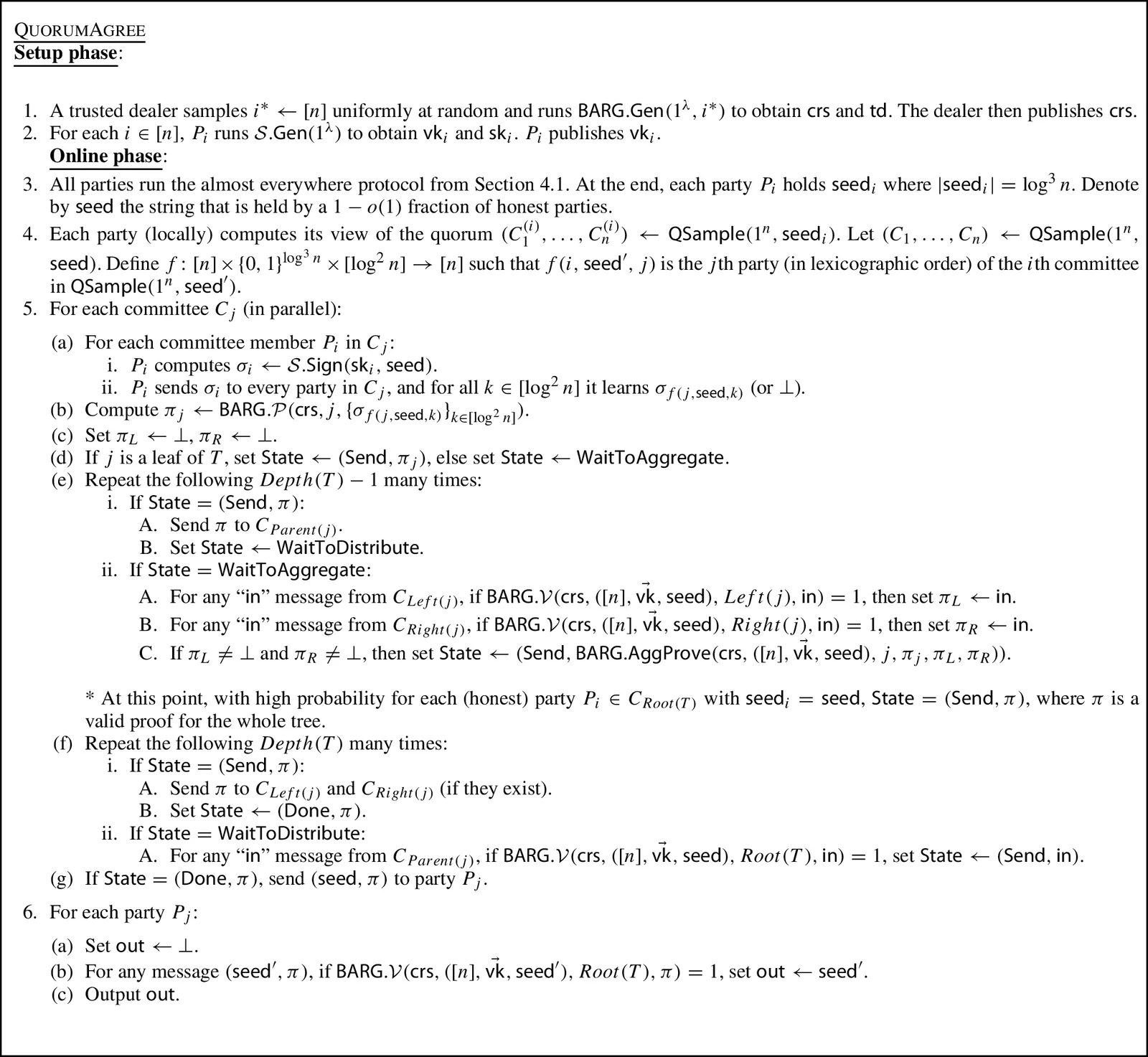

#### Efficiency

##### Lemma 2

The QuorumAgree protocol from Sect. 4.3 terminate after ${\tilde{O}}(1)$ rounds and each party sends $\textsf{poly}(\lambda ,\log n)$ bits overall during the protocol.

##### Proof

By Theorem 5 it is guaranteed that all parties reached the end of step 3 after ${\tilde{O}}(1)$ and until then where each party sent ${\tilde{O}}(1)$ bits. We next analyze the following steps.

*Rounds complexity*: step 5a takes a single round, steps 5e and 5f consist of $O(Depth(T))\subset {\tilde{O}}(1)$ iterations, where each iteration takes a single round. Then, there is an additional round to send the aggregated proof to everyone. So, at total there are ${\tilde{O}}(1)$ rounds, as needed.

*Communication complexity*: consider a single committee. At step 5a each party sends $|\sigma | \in \textsf{poly}(\lambda ,\log n)$ bits to $d \in {\tilde{O}}(1)$ parties. By Theorem 2, during steps 5e and 5f each party sends $\textsf{poly}(\lambda ,\log n)$ bits in each step, and $O(Depth(T)) \subset {\tilde{O}}(1)$. At the last round, each party sends $\textsf{poly}(\lambda ,\log n)$ bits. Finally, since each party is part of exactly *d* committees and $d \in {\tilde{O}}(1)$, it follows that during the entire protocol each party sends at most $\textsf{poly}(\lambda ,\log n)$ bits, as needed. □

#### Security

This section provides the proof of agreement for the above protocol. We show that even if an adversary controls 1/3-ϵ fraction of parties and behaves arbitrarily in their name, the protocol still results with each honest party agreeing on $\textsf{seed}$.

We start by stating the result of our transformation from almost everywhere agreement to everywhere agreement. Note that the transformation and the almost everywhere protocol tolerate a different number of corrupted parties (1/2-ϵ fraction vs. 1/3-ϵ fraction, respectively). Thus, the combined protocol will be secure against 1/3-ϵ fraction of parties. (Still, we decide to state a stronger result below as it might be useful for future works.)

##### Theorem 9

(From almost everywhere to everywhere agreement) For any $\textsf{PPT}$ adversary ${\mathcal {A}}$ that statically corrupts up to 1/2-ϵ fraction of parties, where ϵ is any positive constant, if at the end of step 3 of QuorumAgree at least $1 - O(\log ^{-1} n)$ fraction of (honest) parties hold the same uniformly sampled value, denoted by $\textsf{seed}$, then with probability at least $1 - \textsf{negl}(n) - \textsf{negl}(\lambda )$, at the end of the protocol all (honest) parties outputs $\textsf{seed}$.

Obviously, Theorem 3 follows from Theorem 9 together with the almost everywhere agreement protocol from Theorem 5. The rest of this section is devoted to the proof of Theorem 9.

At a high level, the proof of Theorem 9 consists of two main claims: (1) that the adversary can’t prevent the honest parties from generating a valid proof for $\textsf{seed}$, and (2) that the adversary cannot generate valid proof for a maliciously chosen seed $\textsf{seed}^*$. Consider a $\textsf{PPT}$ adversary ${\mathcal {A}}$ that statically corrupts up to a 1/2-ϵ fraction of parties, where ϵ>0. Denote by ${\mathcal {C}}\subseteq [n]$ the set of corrupted parties. Additionally, we assume (as written in the statement) that at the end of Step 3, at least $1 - O(\log ^{-1} n)$ fraction of (honest) parties hold the same uniformly sampled value, denoted $\textsf{seed}$. Let ${\mathcal {H}}= \{P_i \mid i \in ([n] \setminus {\mathcal {C}}) \wedge \textsf{seed}_i = \textsf{seed}\}$, i.e., ${\mathcal {H}}$ is the set of honest parties that hold the “right” $\textsf{seed}$.

The first claim (Claim 2 below) states that if an honest party is part of committee *j*, then it must hold a valid witness for index *j*, that is, it knows a *w* such that R(j,w)=1. We utilize the following claim that says that every committee contains majority of honest parties that hold the right $\textsf{seed}$. The proof of this claim follows by the construction of the almost everywhere protocol that results with a uniform string which is then fed into our quorum construction.

##### Claim 1

With probability at least $1 - \textsf{negl}(n) - \textsf{negl}(\lambda )$, for all j∈[n], $|{\mathcal {H}}\cap C_j| > |C_j| / 2$.

##### Proof

Recall that with probability at least $1 - \textsf{negl}(n) - \textsf{negl}(\lambda )$, $\textsf{seed}$, the output of the almost everywhere agreement protocol, is sampled uniformly at random and we use it as a seed to generate a quorum (using Theorem 8 with $H = {\mathcal {H}}$). Thus, with probability at least $(1 - \textsf{negl}(n) - \textsf{negl}(\lambda ))(1 - \textsf{negl}(n))$, for each j∈[n],

$$\begin{aligned} |{\mathcal {H}}\cap C_j| = \sum _{z\in C_j} 1\!\!1_{z\in {\mathcal {H}}}&\ge |C_j| \cdot \left( 1- \frac{1}{\log \log n}\right) \cdot \frac{|{\mathcal {H}}|}{n} \\&\ge |C_j| \cdot \frac{(1/2 + \epsilon ) \cdot (1 - o(1)) \cdot n}{n} \\&> |C_j| /2. \end{aligned}$$

□

##### Claim 2

At the end of Step 5a, with probability at least $1 - \textsf{negl}(n) - \textsf{negl}(\lambda )$, for every i,j∈[n], if $P_i \in {\mathcal {H}}\cap C_j$, then party $P_i$ holds $\{\sigma _{f(j, \textsf{seed}, k)}\}_{k \in [\log ^2 n]}$ such that $R(j, \{\sigma _{f(j, \textsf{seed}, k)}\}_{k \in [\log ^2 n]}) = 1$ (see Definition 3).

##### Proof

By description of the protocol, if $P_i \in {\mathcal {H}}\cap C_j$, then the size of the set

$$\begin{aligned} \{k\in [\log ^2 n] \mid \textsf{Verify}(\textsf{vk}_{f(j, \textsf{seed}, k)}, \textsf{seed}, \sigma _{f(j, \textsf{seed}, k)}) = 1\} \end{aligned}$$

is at least $|{\mathcal {H}}\cap C_j|$. Claim 1 implies that $|{\mathcal {H}}\cap C_j| > |C_j| / 2$ and so party $P_i$ holds a valid witness. □

From this point on, it is convenient to assume that for all j∈[n], $|C_j \cap {\mathcal {H}}| > |C_j| / 2$ (which holds w.h.p due to Claim 1). In the next claim, we show that the honest parties aggregate the proof successfully. For simplicity of notation, let $X_\textsf{seed}= ([n], \vec {\textsf{vk}}, \textsf{seed})$.

##### Claim 3

Let $T_j$ be the subtree where *j* is its root. After ℓ iterations of step 5e, for each j∈[n]:

1. If $Depth(T_j) = \ell + 1$, then $\textsf{State} = (\textsf{Send},\pi )$ and ${\mathcal {V}}(\textsf{crs}, X_\textsf{seed}, j, \pi ) = 1$.
2. If $$Depth(T_j) < \ell + 1$$, then $$\textsf{State} = \textsf{WaitToDistribute}$$.
3. If $$Depth(T_j) > \ell + 1$$, then $$\textsf{State} = \textsf{WaitToAggregate}$$.

##### Proof

We prove by induction on ℓ, the number of executed iterations of step 5e.

- Base case: For ℓ=0 and for all j∈[n], *j* is a leaf of *T*. Then,
  1. If $Depth(T_j) = 1$: By step 5d, $\textsf{State} = (\textsf{Send},\pi )$, where $\pi = {\mathcal {P}}({\textsf{crs}, }j, \{\sigma _{f(j, \textsf{seed}, k)}\}_{k \in [\log ^2 n]})$. By the correctness property of the BARG (Definition 1) and by Claim 2, it follows that ${\mathcal {V}}(\textsf{crs}, X_\textsf{seed}, j, \pi ) = 1$.
  2. It is impossible that $Depth(T_j) < 1$, since for every *j*, $Depth(T_j) \ge 1$.
  3. If $Depth(T_j) > 1$: By step 5d, $\textsf{State} = \textsf{WaitToAggregate}$.
- Inductive step: Assume that the claim is true for $0, \dots , \ell - 1$, and we prove it for ℓ. For j∈[n]:
  1. If $$Depth(T_j) = \ell + 1$$, then before the start of the ℓth iteration of step 5e, $$Depth(T_j) > (\ell - 1) + 1$$, so by the inductive assumption, $$\textsf{State} = \textsf{WaitToAggregate}$$. W.l.o.g. assume that the left child sub-tree depth is ℓ and the right child sub-tree depth is $$\ell ' \le \ell $$. Due to the inductive assumption, at the ℓth (resp. $\ell^{\prime }$th) iteration of step 5e, the state of *Left*(*j*) (resp. *Right*(*j*)) was $$(\textsf{Send},\pi )$$, where $${\mathcal {V}}(\textsf{crs}, X_\textsf{seed}, Left(j)$$ (resp. *Right*(*j*)),π)=1. So, from the protocol’s description, $$\textsf{State} = (\textsf{Send},\pi = \textsf{AggProve}(\textsf{crs}, X_\textsf{seed}, j, \pi _j, \pi _L, \pi _R))$$, such that $${\mathcal {V}}(\textsf{crs}, X_\textsf{seed}, j, \pi ) = 1$$.
  2. If $Depth(T_j) < \ell + 1$ there are two cases. First, if $Depth(T_j) < \ell$, we can use the inductive assumption directly. Second, if $Depth(T_j) = \ell$, by the inductive assumption, before the ℓth iteration of step 5e, $\textsf{State} = (\textsf{Send},\pi )$. By the protocol’s description, in the ℓth iteration of step 5e, the state changes to $\textsf{WaitToDistribute}$.
  3. If $Depth(T_j) > \ell + 1$, by the inductive assumption, before the ℓth iteration of step 5e, $\textsf{State} = \textsf{WaitToAggregate}$. W.l.o.g. assume that the left child sub-tree depth is $Depth(T_j) - 1 \ge \ell + 1$. From the inductive assumption, it follows that the left child ends the ℓth iteration of step 5e in state $\textsf{WaitToAggregate}$ or $\textsf{Send}$, so *j* has not yet updated $\pi _L$. □

A corollary of Claim 3 is that the state of all nodes at the end of the iterations of step 5e is as expected.

##### Corollary 2

After the Depth(T)-1 iterations of step 5e, the state of *Root*(*T*) is $(\textsf{Send},\pi )$, where ${\mathcal {V}}(\textsf{crs}, X_\textsf{seed}, Root(T), \pi ) = 1$. Additionally, the state of all other nodes is $\textsf{WaitToDistribute}$.

Now, similarly, we prove that each committee will learn the right proof attesting to the validity of $\textsf{seed}$ w.r.t *T*.

##### Claim 4

Let $T_j$ be the subtree with *j* at its root. After the ℓ iterations of step 5f, for each j∈[n]:

1. If $Depth(T_j) = Depth(T) - \ell$, then $\textsf{State} = (\textsf{Send},\pi )$, where ${\mathcal {V}}(\textsf{crs}, X_\textsf{seed}, Root(T), \pi ) = 1$.
2. If $$Depth(T_j) < Depth(T) - \ell $$, then $$\textsf{State} = \textsf{WaitToDistribute}$$.
3. If $Depth(T_j) > Depth(T) - \ell$, then $\textsf{State} = (\textsf{Done},\pi )$, where ${\mathcal {V}}(\textsf{crs}, X_\textsf{seed}, Root(T), \pi ) = 1$.

##### Proof

By induction on ℓ:

- Base case: For ℓ=0, and for all j∈[n]:
  1. If $Depth(T_j) = Depth(T)$, then since *j* is the root of *T*, the claim follows from Corollary 2.
  2. If $Depth(T_j) < Depth(T)$, then *j* is not the root of *T* and so the claim follows from Corollary 2.
  3. It is impossible that $Depth(T_j) > Depth(T)$ since for every *j*, $Depth(T_j) \le Depth(T)$.
- Inductive step: Assume that the claim is true for $0, \dots , \ell - 1$, and we will prove it for ℓ. For j∈[n]:
  1. If $Depth(T_j) = Depth(T) - \ell$, by the inductive assumption, before the ℓth iteration of step 5f, the state of *j* is $\textsf{WaitToDistribute}$, and the state of *Parent*(*j*) is $(\textsf{Send},\pi )$, where ${\mathcal {V}}(\textsf{crs}, X_\textsf{seed}, Root(T), \pi ) = 1$. Since there is at least one honest party in each committee (actually, there is an honest majority) and by description of step 5f, at the end of the ℓth iteration of step 5f, the state of *j* will be $(\textsf{Send},\pi )$, as needed.
  2. If $Depth(T_j) < Depth(T) - \ell$, from the inductive assumption, before the ℓth iteration of step 5f, the state of *j* and his parent is $\textsf{WaitToDistribute}$. So, by description of step 5f, at the end of the ℓth iteration of step 5f, the state of *j* will be $\textsf{WaitToDistribute}$, as needed.
  3. If $Depth(T_j) > Depth(T) - \ell$ there are two cases. First, if $Depth(T_j) > Depth(T) - \ell + 1$, we use the inductive assumption directly. Second, if $Depth(T_j) = Depth(T) - \ell + 1$, by the inductive assumption, before the ℓth iteration of step 5f, $\textsf{State} = (\textsf{Send},\pi )$, where ${\mathcal {V}}(\textsf{crs}, X_\textsf{seed}, Root(T), \pi ) = 1$. Thus, by the protocol’s description, during the ℓth iteration of step 5f, the state changes to $(\textsf{Done},\pi )$. □

A corollary of Claim 4 is that the state of all nodes at the end of the iterations of step 5f. is as expected.

##### Corollary 3

After the *Depth*(*T*) iterations of step 5f the state of all nodes j∈[n] is $(\textsf{Done},\pi )$. Additionally, ${\mathcal {V}}(\textsf{crs}, X_\textsf{seed}, Root(T), \pi ) = 1$.

Next, we conclude that with high probability, each honest party will receive *at least once* the right $\textsf{seed}$ along with a valid proof.

##### Lemma 3

With probability at least $1 - \textsf{negl}(n) - \textsf{negl}(\lambda )$, at step 6b, every (honest) party sets $\textsf{out} = \textsf{seed}$, where $\textsf{seed}$ is the value that is outputted by the almost-everywhere agreement sub-protocol.

##### Proof

By Corollary 3, each committee’s state is $(\textsf{Done},\pi )$, where ${\mathcal {V}}(\textsf{crs}, X_\textsf{seed}, Root(T), \pi ) = 1$. Together with Claim 1, every party receives at least once the right $\textsf{seed}$ and a valid proof. Thus, after step 6b, each party will update $\textsf{out}$ with $\textsf{seed}$. □

The above lemma stands for the protocol’s “correctness”. In other words, we proved that the adversary cannot prevent from any honest party from receiving the right $\textsf{seed}$ along with a valid proof for it. However, we have not yet proved that the adversary cannot generate another valid proof for a wrong $\textsf{seed}$, which is also undesirable. For simplicity, assume that the adversary is given free of charge full control over unknowledgeable honest parties, i.e., parties with $\textsf{seed}_i \ne \textsf{seed}$ (after step 6). Notice that this group is of size *o*(*n*), so this does not add significant power to the attacker.

We show that if there exists an adversary that generates a valid proof for a maliciously chosen seed, then we can build another adversary that wins the unforgeability game of the signature scheme (Sect. 3.2.1) with only slightly lower probability. Our adversary uses the BARG scheme knowledge extractor from Definition 1 which, to recall, is an efficient procedure that receives valid proofs for some $\textsf{seed}'$ and extracts (w.h.p.) the witness associated with some committee $C_{i^*}$, where the index $i^*$ is supplied during setup.

##### Claim 5

Assume a $\textsf{PPT}$ adversary ${\mathcal {A}}'$ that at step 6b of the protocol can generate a pair $(\textsf{seed}', \pi ')$ where $\textsf{seed}' \ne \textsf{seed}$ and $\Pr [{\mathcal {V}}(\textsf{crs}, X_{\textsf{seed}'}, Root(T), \pi ') = 1] \ge p(n)$. Then, there is another $\textsf{PPT}$ adversary ${\mathcal {A}}$ such that $\Pr [\textsf{ExUnfg}_{{\mathcal {S}},{\mathcal {A}}}(\lambda ) = 1 ] \ge p(n)/2n$, where the game $\textsf{ExUnfg}_{{\mathcal {S}}, {\mathcal {A}}}(\lambda )$ is defined in Sect. 3.2.1.

##### Proof

Let ${\mathcal {E}}$ be the knowledge extractor of the BARG scheme (Definition 1). We build ${\mathcal {A}}$ as follows:

1. ${\mathcal {A}}$ chooses k=n, and receives $\{\textsf{vk}_i\}_{i \in [n]}$ from the experiment.
2. ${\mathcal {A}}$ samples $i^*\leftarrow [n]$ and runs $(\textsf{crs}, \textsf{td}) \leftarrow \textsf{BARG}.\textsf{Gen}(1^\lambda , i^*)$.
3. ${\mathcal {A}}$ sends $\textsf{crs}$ and $\{\textsf{vk}_i\}_{i \in [n]}$ to ${\mathcal {A}}'$.
4. ${\mathcal {A}}'$ chooses the set of corrupted parties ${\mathcal {C}}$ and sends ${\mathcal {C}}$ to ${\mathcal {A}}$. ${\mathcal {A}}$ sets ${\mathcal {C}}$ as its corrupted set, receives $\{\textsf{sk}_i\}_{i \in {\mathcal {C}}}$ from the experiment, and forwards it to ${\mathcal {A}}'$.
5. ${\mathcal {A}}$ and ${\mathcal {A}}'$ run together the QuorumAgree protocol up to the end of step 6b, where ${\mathcal {A}}$ simulates $[n] \setminus {\mathcal {C}}$. When it needs to sign a message *m*, the experiment signs it for him. At the end, ${\mathcal {A}}'$ sends $(\textsf{seed}', \pi ')$ to ${\mathcal {A}}$.
6. ${\mathcal {A}}$ sends $(X_{\textsf{seed}'}, \textsf{td}, i^*, Root(T), \pi ')$ to ${\mathcal {E}}$, and it answers with $\{\sigma _{f(i^*, \textsf{seed}', j)}\}_{j \in [\log ^2 n]}$.
7. For each $j^* \in \{f(i^*, \textsf{seed}', j)\}_{j \in [\log ^2 n]}$, if $j^* \in [n] \setminus {\mathcal {C}}$ and $\textsf{Verify}(\textsf{vk}_{j^*}, \textsf{seed}', \sigma _{j^*}) = 1$, then ${\mathcal {A}}$ outputs $(j^*, \textsf{seed}', \sigma _{j^*})$.
8. $${\mathcal {A}}$$ outputs $$(\bot , \bot , \bot )$$.

To analyze the above reduction, we define the following events:

- $$\underline{\hbox {Event }E_{\textsf{AcceptProof}}}$$: $${\mathcal {V}}(\textsf{crs}, X_{\textsf{seed}'}, Root(T), \pi ') = 1$$.
- $$\underline{\hbox {Event }E_{\textsf{EnoughValidSigs}}}$$: $$R_{{\mathcal {S}}, f, \vec {\textsf{vk}}, \textsf{seed}'}(i^*, \{\sigma _{f(i^*, \textsf{seed}', j)}\}_{j \in [\log ^2 n]}) = 1$$. (See Definition 3.)
- $\underline{\hbox {Event }E_{\textsf{HonestMajority}}^{(i)}}$: $|C_i \cap ([n] \setminus {\mathcal {C}})| > \log ^2 n/2$, where $C_i$ the *i*th committee in the quorum $\textsf{QSample}(n, \textsf{seed}')$.
- $\underline{\hbox {Event }E_1}$: The experiment outputs 1.

First, recall that every committee size is exactly $\log ^2 n$, independently of $\textsf{seed}'$. By the definition of $R_{{\mathcal {S}}, f, \vec {\textsf{vk}}, \textsf{seed}'}$ if $E_{\textsf{AcceptProof}}$ happens, then

$$\begin{aligned} \sum _{j \in [\log ^2 n]} \textsf{Verify}(\textsf{vk}_{f(i^*, \textsf{seed}', j)}, \textsf{seed}, \sigma _{f(i^*, \textsf{seed}', j)}) > \log ^2 n / 2. \end{aligned}$$

This means that $\Pr [E_{\textsf{EnoughValidSigs}}\wedge E_{\textsf{HonestMajority}}^{(i^*)} \wedge \lnot E_1] = 0$. From the “no terrible quorum” property (see Definition 2), we know that $\Pr [\exists i \in [n] :E_{\textsf{HonestMajority}}^{(i)}] = 1$. Since ${\mathcal {A}}'$ is unaware of $i^*$, $\Pr [E_{\textsf{HonestMajority}}^{(i^*)}] \ge 1/n$, and also $E_{\textsf{HonestMajority}}^{(i^*)}$ and $E_{\textsf{AcceptProof}}$ are independent events.^7^ Next, from the claim requirement it follows that $\Pr [E_{\textsf{AcceptProof}}] \ge p(n)$, and from the “somewhere argument of knowledge” property of the BARG scheme we conclude that

$$\begin{aligned}&\Pr [E_{\textsf{EnoughValidSigs}}\mid E_{\textsf{AcceptProof}}\wedge E_{\textsf{HonestMajority}}^{(i^*)}] \ge 1/2. \end{aligned}$$

Finally,

$$\begin{aligned}&{\Pr [E_1]} \ge \Pr \left[ E_{\textsf{EnoughValidSigs}}\wedge E_{\textsf{HonestMajority}}^{(i^*)}\right] \\&\quad \ge \Pr \left[ E_{\textsf{AcceptProof}}\wedge E_{\textsf{EnoughValidSigs}}\wedge E_{\textsf{HonestMajority}}^{(i^*)}\right] \\&\quad =\Pr \left[ E_{\textsf{HonestMajority}}^{(i^*)} \wedge E_{\textsf{AcceptProof}}\right] \\&\qquad \cdot \Pr \left[ E_{\textsf{EnoughValidSigs}}\mid E_{\textsf{HonestMajority}}^{(i^*)} \wedge E_{\textsf{AcceptProof}}\right] \\&\quad =\Pr \left[ E_{\textsf{AcceptProof}}\right] \cdot \Pr \left[ E_{\textsf{HonestMajority}}^{(i^*)}\right] \\&\qquad \cdot \Pr \left[ E_{\textsf{EnoughValidSigs}}\mid E_{\textsf{HonestMajority}}^{(i^*)} \wedge E_{\textsf{AcceptProof}}\right] \\&\quad \ge {p(n)/2n}. \end{aligned}$$

□

Next, we show how to use the above reduction to prove that the adversary cannot generate a proof for a wrong seed.

##### Lemma 4

With probability at least $1 - \textsf{negl}(n) - \textsf{negl}(\lambda )$, at step 6b, if (honest) party sets $\textsf{out} = \textsf{seed}$, then $\textsf{seed}$ is the value that defines ${\mathcal {H}}$ (the almost everywhere agreed value).

##### Proof

Assume, by contradiction, that the claim is false. That is, with noticeable probability an honest party sets $\textsf{out} = \textsf{seed}'$ for some $\textsf{seed}'\ne \textsf{seed}$. Then, by Corollary 3, there exists a $\textsf{PPT}$ adversary ${\mathcal {A}}$ that can generate a pair $(\textsf{seed}',\pi ')$ such that $\textsf{seed}' \ne \textsf{seed}$ and for some noticeable function *f*(*n*), $\Pr [{\mathcal {V}}(\textsf{crs}, X_{\textsf{seed}'}, Root(T), \pi ') = 1] \ge f(n)$. By Claim 5, there is another $\textsf{PPT}$ adversary ${\mathcal {A}}'$ that win the PKI unforgeability game of the signature scheme (Sect. 3.2.1) with noticeable probability. This is a contradiction.

□

Finally, Theorem 9 follows by combining Lemmas 3 and 4.

## Data Availability

No datasets were generated or analysed during the current study.

## Footnotes

1 Informally, LWE asks to distinguish noisy linear equations over a finite ring from uniform; see Sect. 3.2 for a formal definition.

2 This is an argument system that allows an efficient prover to compute a non-interactive and publicly verifiable “batch proof” of NP instances, with size smaller than the combined witness length. If any of the instances is false, then no polynomial-time cheating prover must be able to produce an accepting proof.

3 A SNARK is a proof system that enables a prover holding a witness *w* to some public NP statement *x* to convince a verifier that it indeed knows *w* by sending a single message. The proof string is succinct in the sense that it is much shorter than the witness *w*, and knowledge is formalized via an efficient extractor that succeeds extracting *w* from a malicious prover $P^*$ with roughly the same probability that $P^*$ convinces an honest verifier. The work of [22] assumes linear extraction, i.e., where the size of the extractor is linear in the size of the prover.

4 Throughout, the ${\tilde{O}}(\cdot )$ (and $\tilde{\Omega }(\cdot )$) notation hides poly-logarithmic factors in the number of parties *n*, consistently with the notation paragraph above.

5 Since our constructions rely on a public-key infrastructure it is easy to “implement” these properties (e.g., by using authenticated encryption).

6 As mentioned, the idea to define the quorums via fixed shifts of a single “sufficiently random” committee is due to [19]. However, there the randomness condition on the single committee is weaker and also they did not consider the “no terrible quorum” property.

7 Recall that for independent events *E* and $E^{\prime }$, $$\Pr [E \wedge E'] = \Pr [E] \cdot \Pr [E']$$.
